# Response to the letter to the editor: Stress-induced hyperglycemia and expression of glucose cell transport genes in skeletal muscle of critically ill patients: a cross-sectional study

**DOI:** 10.20945/2359-4292-2025-0356

**Published:** 2025-10-08

**Authors:** Priscila Bellaver, Daisy Crispim, Lílian Rodrigues Henrique, Cristiane Bauermann Leitão, Ariell Freires Schaeffer, Tatiana Helena Rech, Diego Paluszkiewicz Dullius

**Affiliations:** 1 Programa de Pós-Graduação em Ciências Médicas: Endocrinologia, Universidade Federal do Rio Grande do Sul, Porto Alegre, RS, Brasil; 2 Unidade de Terapia Intensiva, Hospital de Clínicas de Porto Alegre, Porto Alegre, RS, Brasil; 3 Faculdade de Medicina, Universidade Federal do Rio Grande do Sul, Porto Alegre, RS, Brasil; 4 Divisão de Cirurgia Plástica, Hospital de Clínicas de Porto Alegre, Porto Alegre, RS, Brasil; 5 Grupo de Diabetes e Metabolismo, Centro de Pesquisa Clínica e Divisão Endocrinologia, Hospital de Clínicas de Porto Alegre, Porto Alegre, RS, Brasil; 6 Departamento de Medicina Interna, Universidade Federal do Rio Grande do Sul, Porto Alegre, RS, Brasil

Dear Editor,

We thank Dr. Rahim and Dr. Khurshid for their thoughtful comments regarding our study entitled “Stress-induced hyperglycemia and expression of glucose cell transport genes in skeletal muscle of critically ill patients: a cross-sectional study”^(1)^. Their insights enrich the ongoing discussion about the metabolic alterations that occur during critical illness. The authors point out several factors that could confound the interpretation of insulin receptor substrate 1 (*IRS1*) downregulation in the context of stress-induced hyperglycemia. We would like to take this opportunity to address their concerns.

We agree that both exogenous corticosteroids and hepatic dysfunction significantly influence glucose metabolism. In our study, 42% of patients received corticosteroids at some point during their ICU course, however biopsies were taken within the first 24 hours of intensive care unit (ICU) admission, before a significant corticosteroid effect. It is important to note that several factors contribute both to augmented glucose production and insulin resistance in critically ill patients, some of them not routinely quantified, such as systemic inflammation ^(2)^. In fact, disturbances in glycemic control due to hepatic dysfunction in the ICU are uncommon outside the setting of acute liver failure. In our study, two patients presented with cirrhosis Child A, as coagulation disorders were excluded. In the critically ill patient, hyperglycemia primarily occurs due to an excess of counter-regulatory hormones, as well as elevated levels of circulating cytokines acting on the liver and promoting hepatic gluconeogenesis. The use of corticosteroids, a known contributor to hyperglycemia, is extremely common in critically ill patients ^(3)^ and its effects in skeletal muscle gene expression may be implicated on the underlying mechanisms of stress-induced hyperglycemia. Despite these potential confounders, our sample size was not large enough to allow for adjustments. We recognize this limitation and suggest that future studies should either control for or stratify by variables influencing glucose control to better isolate the contribution of hyperglycemia itself to the molecular changes in insulin signaling pathways.

We had previously reported on the downregulated of INSR during stress-induced hyperglycemia ^(4)^. Our present study focused on *IRS1, IRS2, SLC2A1*, and *SLC2A4* due to their well-established roles in glucose transport and insulin signaling, especially in skeletal muscle, which is a known target of the sepsis-induced inflammation. We recognize that insulin resistance is a multifactorial process involving an intricate regulatory network, including the skeletal muscle. To point out a single gene as the culprit is challenging. Indeed, RNA sequencing would provide a more comprehensive understanding of these complex dialogue involving the muscle, the liver, the pancreas, and the immunological system during critical illness. Our findings should be viewed as a targeted investigation within a broader mechanistic landscape.

Despite its limitations, the major positive aspect of our study is that it offers initial insights into changes in gene expression related to stress-induced hyperglycemia, which is a well-established risk factor for worse outcomes in critical illness ^(5)^. We believe that incorporating broader molecular profiling, inflammatory markers quantification, and genetic data will be relevant in future investigations to better elucidate the mechanisms underlying insulin resistance in critical illness.

## Data Availability

datasets related to this article will be available upon request to the corresponding author.

## References

[1] Bellaver P, Henrique LR, Schaeffer AF, Dullius DP, Crispim D, Leitão CB (2025). Stress-induced hyperglycemia and expression of glucose cell
transport genes in skeletal muscle of critically ill patients: a
cross-sectional study. Arch Endocrinol Metab.

[2] Song G, Liu X, Lu Z, Guan J, Chen X, Li Y (2025). Relationship between stress hyperglycaemic ratio (SHR) and
critical illness: a systematic review. Cardiovasc Diabetol.

[3] Haan BJ, Blackmon SN, Cobb AM, Cohen HE, DeVier MT, Perez MM (2024). Corticosteroids in critically ill patients: A narrative
review. Pharmacotherapy.

[4] Bellaver P, Schaeffer AF, Dullius DP, Crispim D, Leitão CB, Rech TH. (2023). Association between diabetes and stress-induced hyperglycemia
with skeletal muscle gene expression of INSR in critically ill patients: A
prospective cohort study. Diabetes Metab.

[5] Bellaver P, Schaeffer AF, Dullius DP, Viana MV, Leitão CB, Rech TH. (2019). Association of multiple glycemic parameters at intensive care
unit admission with mortality and clinical outcomes in critically ill
patients. Sci Rep.

